# Intranasal PAMAM-G3 scavenges cell-free DNA attenuating the allergic airway inflammation

**DOI:** 10.1038/s41420-024-01980-x

**Published:** 2024-05-02

**Authors:** Xiumin Chen, Changhui Chen, Zhaoxu Tu, Zeling Guo, Tong Lu, Jian Li, Yihui Wen, Dehua Chen, Wenbin Lei, Weiping Wen, Hang Li

**Affiliations:** 1 Department of Otorhinolaryngology, the First Affiliated Hospital of Sun Yat-sen University, Guangzhou, China; 2 Otorhinolaryngology Hospital, Sun Yat-sen University, Guangzhou, China; 3 Department of Otorhinolaryngology, the Sixth Affiliated Hospital of Sun Yat-sen University, Guangzhou, China; 4 Department of Otorhinolaryngology, Guangxi Hospital Division of the First Affiliated Hospital, Sun Yat-sen University, Nanning, China

**Keywords:** Prognostic markers, Cell death and immune response

## Abstract

Allergic airway inflammation (AAI), including allergic rhinitis (AR) and allergic asthma, is driven by epithelial barrier dysfunction and type 2 inflammation. However, the underlying mechanism remains uncertain and available treatments are constrained. Consequently, we aim to explore the role of cell-free DNA (cfDNA) in AAI and assess the potential alleviating effects of cationic polymers (CPs) through cfDNA elimination. Levels of cfDNA were evaluated in AR patients, allergen-stimulated human bronchial epithelium (BEAS-2B cells) and primary human nasal epithelium from both AR and healthy control (HC), and AAI murine model. Polyamidoamine dendrimers-generation 3 (PAMAM-G3), a classic type of cationic polymers, were applied to investigate whether the clearance of cfDNA could ameliorate airway epithelial dysfunction and inhibit AAI. The levels of cfDNA in the plasma and nasal secretion from AR were higher than those from HC (*P* < 0.05). Additionally, cfDNA levels in the exhaled breath condensate (EBC) were positively correlated with Interleukin (IL)-5 levels in EBC (*R* = 0.4191, *P* = 0.0001). Plasma cfDNA levels negatively correlated with the duration of allergen immunotherapy treatment (*R* = −0.4297, *P* = 0.006). Allergen stimulated cfDNA secretion in vitro (*P* < 0.001) and in vivo (*P* < 0.0001), which could be effectively scavenged with PAMAM-G3. The application of PAMAM-G3 inhibited epithelial barrier dysfunction in vitro and attenuated the development of AAI in vivo. This study elucidates that cfDNA, a promising biomarker for monitoring disease severity, aggravates AAI and the application of intranasal PAMAM-G3 could potentially be a novel therapeutic intervention for AAI.

**Figure:**
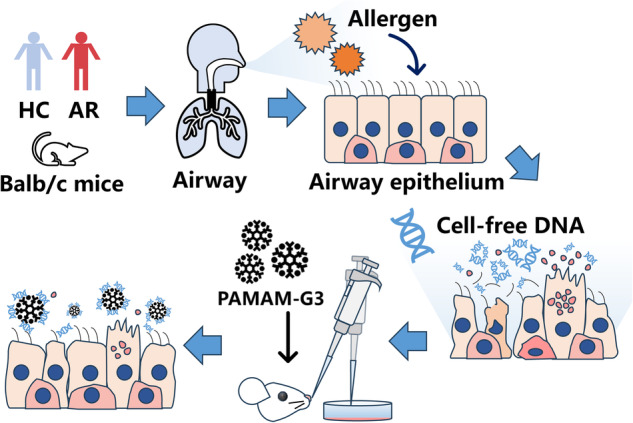
Allergen stimulates the secretion of cell-free DNA (cfDNA) in both human and mouse airway. Intranasal polyamidoamine dendrimers-generation 3 (PAMAM-G3) scavenges cfDNA and alleviates allergic airway inflammation.

Allergen stimulates the secretion of cell-free DNA (cfDNA) in both human and mouse airway. Intranasal polyamidoamine dendrimers-generation 3 (PAMAM-G3) scavenges cfDNA and alleviates allergic airway inflammation.

## Introduction

The incidence of allergic airway inflammation (AAI), including allergic rhinitis (AR) and allergic asthma, is on the rise globally [1, 2], posing a significant public health challenge [3, 4]. AAI is characterized by immunoglobulin E (IgE)-mediated reactions triggered by inhaled allergens, such as house dust mite (HDM) and pollen, and is closely associated with epithelial barrier dysfunction and type 2 inflammation [5, 6]. Nevertheless, the precise underlying mechanism of AAI remains uncertain. Up to now, the primary therapeutic approaches for AR encompass symptomatic treatments and allergen immunotherapy (AIT), which is the only etiological and potentially curative therapy [1]. However, long-term treatment and its associated high cost often lead to inadequate patient compliance [7], which may result in treatment failure [8, 9]. Consequently, novel therapeutic strategies for AR and dependable monitoring biomarkers for AIT are imperative [1, 10].

Cell-free DNA (cfDNA), is predominantly composed of double helix DNA fragments and originates from various sources, such as expelled nuclei from erythroid precursors, mitochondrial DNA, and extracellular traps [11]. It can be detected in a wide range of body fluids, such as plasma, urine, and sputum [12]. The immunostimulatory properties of DNA were discovered half a century ago [13]. Subsequent studies have increasingly demonstrated the association between cfDNA and numerous immune disorders, such as systemic lupus erythematosus (SLE) [14, 15], rheumatoid arthritis (RA), and transplantation rejection [16, 17]. Consequently, researchers have focused on investigating therapeutic strategies derived from cfDNA [18, 19]. Recently, several studies revealed that cfDNA [12], including double-stranded DNA [20] (dsDNA) in nasal-lavage samples, neutrophil extracellular traps (NETs) [20–22] in bronchoalveolar lavage fluid (BALF) and sputum, and eosinophil extracellular traps (EETs) in BALF and endobronchial biopsy specimens [22, 23], was associated with asthma. These studies propose that cfDNA plays a role in both physiological and pathological processes related to inflammation and autoimmunity [24]. Notably, cfDNA has been clinically applied in liquid biopsies, specifically in the examination of cfDNA in plasma, such as noninvasive prenatal testing, cancer liquid biopsies, and monitoring graft dysfunction [25]. Moreover, recent studies show that the clearance of cfDNA from plasma could alleviate inflammation in SLE [14] and RA [18]. Given its potential role in various diseases and its existence in a wide variety of body fluids, cfDNA demonstrates promising prospects for widespread application. However, its role in the pathogenesis of AR remains unknown and the clinical treatments targeting cfDNA are lacking.

Cationic polymers (CPs) are a class of biomaterials known for their ability to effectively bind with nucleic acids. These polymers possess flexible properties, can be easily synthesized, and exhibit high efficiency in gene delivery [26]. CPs are not only potentially useful for delivering therapeutic agents but also for scavenging cfDNA in autoimmune diseases [27]. The properties of CPs could be adjusted as their structures are changed, which provides potentially wide applications [28]. Polyamidoamine (PAMAM) dendrimers, a type of CPs, are hyperbranched polymers with 32 surface amine groups that enable effective binding with nucleic acids. Therefore, they have been utilized in gene transfection, drug delivery, biomimetic artificial proteins and others [29, 30]. PAMAM dendrimers have been the subject of anti-inflammatory research in SLE [31, 32], corneal inflammation [33], and obesity-associated chronic inflammation [34]. However, their role in AAI remains unexplored.

In this study, we sought to investigate the involvement of cfDNA in AR and the potential application of intranasal PAMAM dendrimers in AAI.

## Results

### cfDNA is related to allergic airway inflammation

**Fig. 1:**
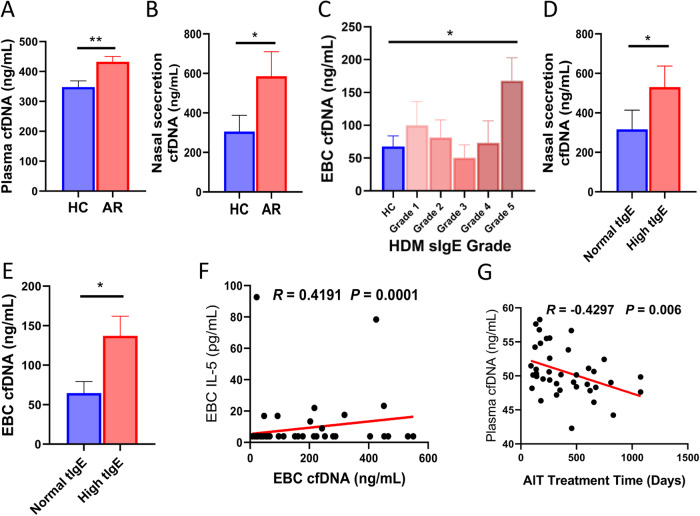
Cell-free DNA is related to allergic rhinitis (AR). The concentrations of cfDNA in the plasma (**A**) and nasal secretion (**B**) of allergic rhinitis (AR) participants and healthy control (HC) participants were evaluated. (**C**) cfDNA levels in the exhaled breath condensate (EBC) of different house dust mite (HDM)-specific IgE (sIgE) grade groups. cfDNA levels in the nasal secretion (**D**) and EBC (**E**) between high total IgE (tIgE) group and normal tIgE group. **F** The correlation between cfDNA and Interleukin (IL)-5 concentration in EBC. **G** The correlation between cfDNA levels in the plasma and allergen immunotherapy (AIT) treatment time. Data was expressed as means ± SEM. *P < 0.05, **P < 0.01.

As we mentioned above, cfDNA is associated with human asthma [20–23]. To investigate the role of cfDNA in AR, we evaluated the levels of cfDNA in the plasma, nasal secretion, and EBC from both HC and AR participants, and detected the concentrations of type 2 immune response cytokines, including IL-4 and IL-5. We discovered that the cfDNA levels from AR participants were higher than those from HC participants both in the plasma (Fig. 1A, *P* = 0.003) and nasal secretion (Fig. 1B, *P* = 0.02). cfDNA concentrations in the EBC from HDM-sIgE grade 5 group were higher than those from HC group (Fig. 1C, *P* = 0.01). As shown in eFigure 2, the correlation between cfDNA levels in EBC and HDM-sIgE level in plasma was analyzed (*R* = 0.2096, *P* = 0.02). Besides, when the participants were divided into high tIgE group and normal tIgE group [35], the high tIgE group has higher levels of cfDNA in both nasal secretion and EBC than the normal tIgE group (Fig. 1D, *P* = 0.048, Fig. 1E, *P* = 0.02). The levels of IL-5 in EBC were positively correlated with the levels of cfDNA in EBC (Fig. 1F, *R* = 0.4191 *P* = 0.0001). The levels of IL-4 were below the detection limit. Levels of cfDNA were also evaluated in AR patients who were receiving AIT treatment, and the results showed that cfDNA concentrations were negatively correlated with the treatment time of AIT (Fig. 1G, *R* = −0.4297, *P* = 0.006).

### Allergen exposure stimulated the secretion of cfDNA in human airway epithelial cells in vitro

**Fig. 2:**
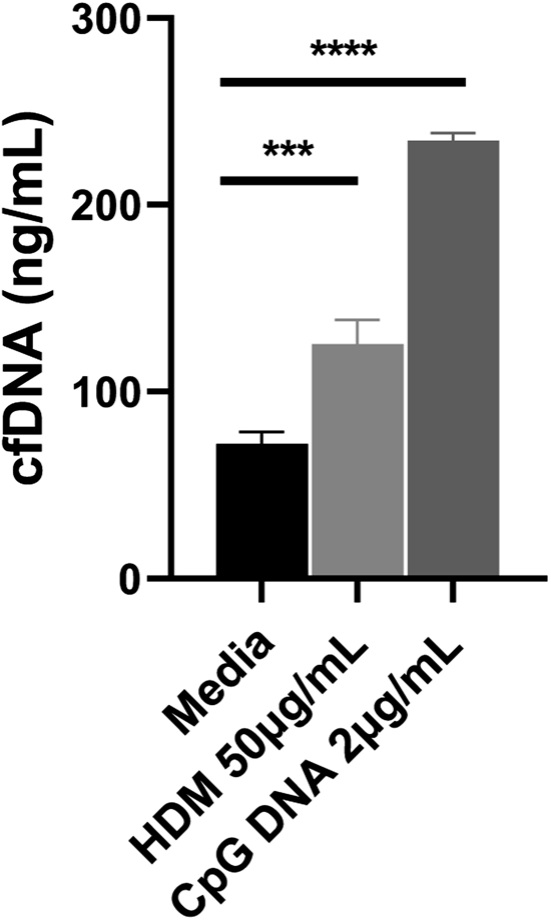
HDM stimulated the secretion of cfDNA in human airway epithelial cells in vitro. Compared to media, cfDNA of HDM group was significantly higher (*P* < 0.001). CpG DNA was set as a positive control. Data was expressed as means ± SEM. ***P < 0.001, ****P < 0.0001.

To explore whether allergen stimulation could cause the secretion of cfDNA in human airway epithelium, we exposed BEAS-2B cells with HDM in vitro. After 72 h exposure, the cfDNA concentrations of HDM group were significantly higher than those of control group (Fig. 2, *P* = 0.0004). The results showed that the airway epithelium exposed to allergen would subsequently release a mass of cfDNA.

### PAMAM-G3 scavenged cfDNA and inhibited the epithelial barrier dysfunction of epithelial cells induced by allergen exposure in vitro

**Fig. 3:**
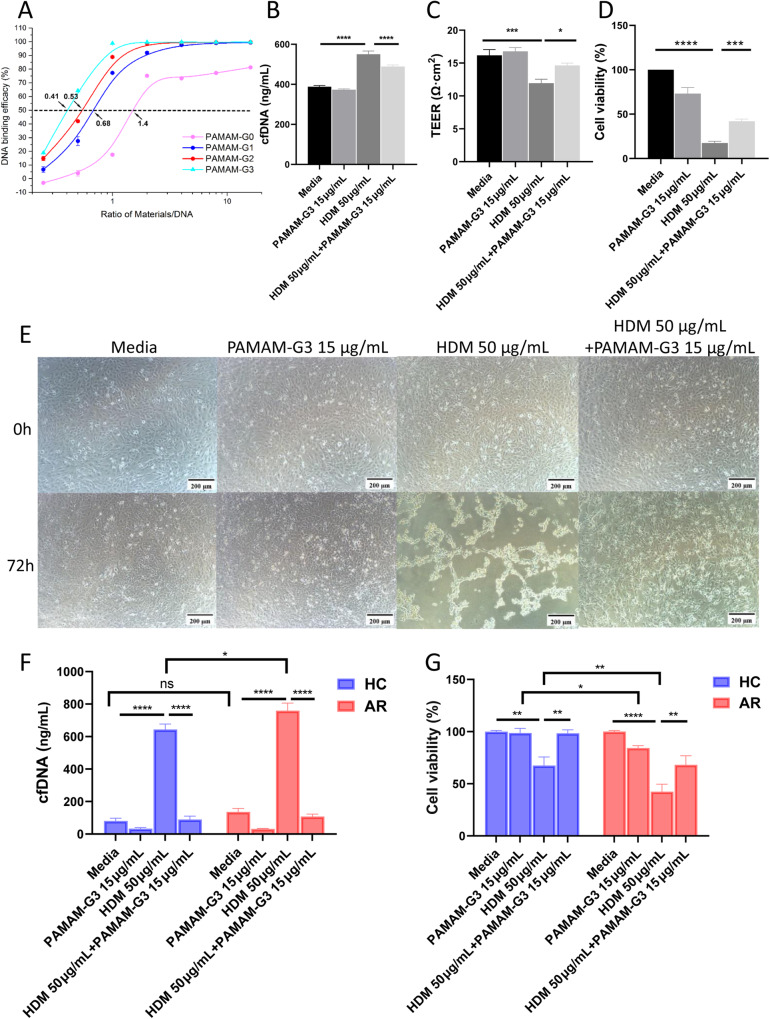
Polyamidoamine dendrimers generation 3 (PAMAM-G3) scavenged cfDNA in vitro. **A** DNA binding efficacy of different generation of PAMAM. **B** PAMAM-G3 reduced cfDNA released by HDM stimulation. **C** Trans-epithelial Electrical Resistance (TEER) of different groups in BEAS-2B cells. **D** Cell Counting Kit-8 (CCK-8) assay of different groups in BEAS-2B cells. **E** The morphology of BEAS-2B cells. **F** cfDNA levels of different groups in primary nasal epithelial cells. **G** CCK-8 assay of different groups in primary nasal epithelial cells. Scale bars, 200 μm. Data was expressed as means ± SEM. *P < 0.05, **P < 0.01, ***P < 0.001, ****P < 0.0001.

To investigate whether CPs could eliminate the secretion of cfDNA and inhibit the epithelium dysfunction following allergen exposure, PAMAM-G3, which is a representative of CPs, was used in this study. As shown in Fig. 3A, PAMAM-G3 is an effective DNA adsorbent compared to other lower generations of PAMAM. PAMAM-G3 significantly decreased the cfDNA released by HDM exposure not only in BEAS-2B (Fig. 3B, *P* < 0.0001) but also in primary human nasal epithelial cells (Fig. 3F, *P* < 0.0001). Furthermore, the application of PAMAM-G3 changed TEER to normal values in BEAS-2B cells (Fig. 3C, *P* = 0.02) and restored viability decrease caused by HDM exposure both in BEAS-2B cells and primary human nasal epithelium cells (Fig. 3D, G). Consistently with the results in BEAS-2B, HDM exposure also stimulated the secretion of cfDNA in primary human nasal epithelial cells (Fig. 3F). Such effect was not limited to atopic nasal epithelial cells. However, compared to HC, nasal epithelium cells from AR participants showed a higher level of cfDNA and lower cell viability after HDM exposure (Fig. 3F, G).

### Intranasal PAMAM-G3 reduced AAI in a murine model

**Fig. 4:**
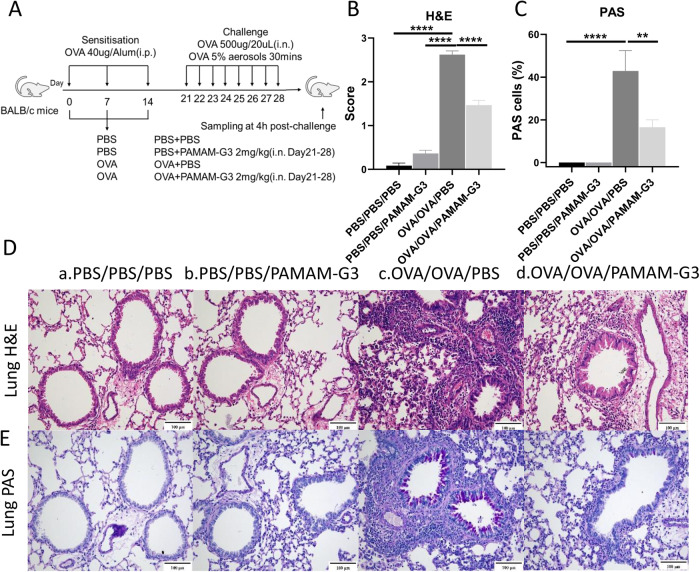
Administration of PAMAM-G3 in a murine model of AAI. **A** The experimental protocol of applying PAMAM-G3 in a murine model of AAI. **B**, **D** Representative Hematoxylin and Eosin (H&E) staining and inflammation score of lung tissues. **C** **E** Representative Periodic Acid-Schiff (PAS) staining and PAS-positive cells score of lung tissues. Scale bars, 100 μm. Data was expressed as means ± SEM. OVA ovalbumin, Alum aluminum hydroxide, PBS phosphate-buffered saline, i.n. intranasal, i.e. intraperitoneal. **P < 0.01, ****P < 0.0001.

**Fig. 5:**
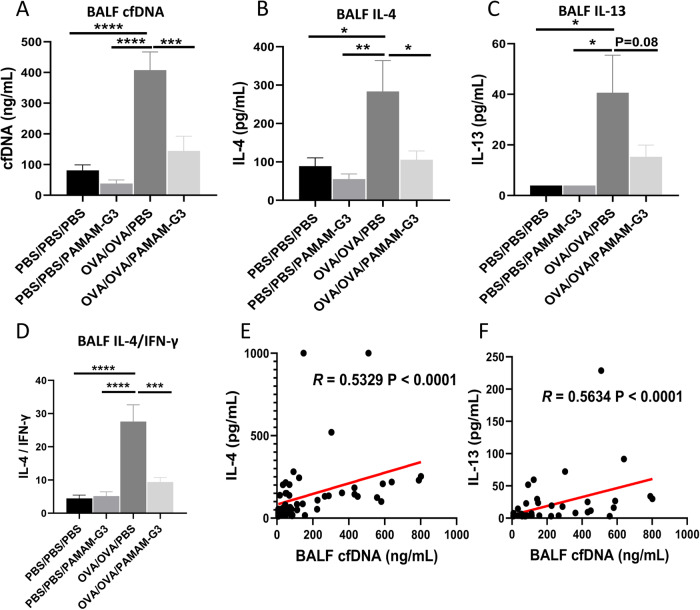
PAMAM-G3 reduced cfDNA and type 2 inflammation in AAI. **A** cfDNA of bronchoalveolar lavage fluid (BALF) in different groups. PAMAM-G3’s effect on IL-4 (**B**), IL-13 (**C**), and the ratio of IL-4 to IFN-γ (**D**) in BALF. cfDNA was positively correlated with IL-4 (**E**) and IL-13 (**F**). Data was expressed as means ± SEM. *P < 0.05, **P < 0.01, ***P < 0.001, ****P < 0.0001.

**Fig. 6:**
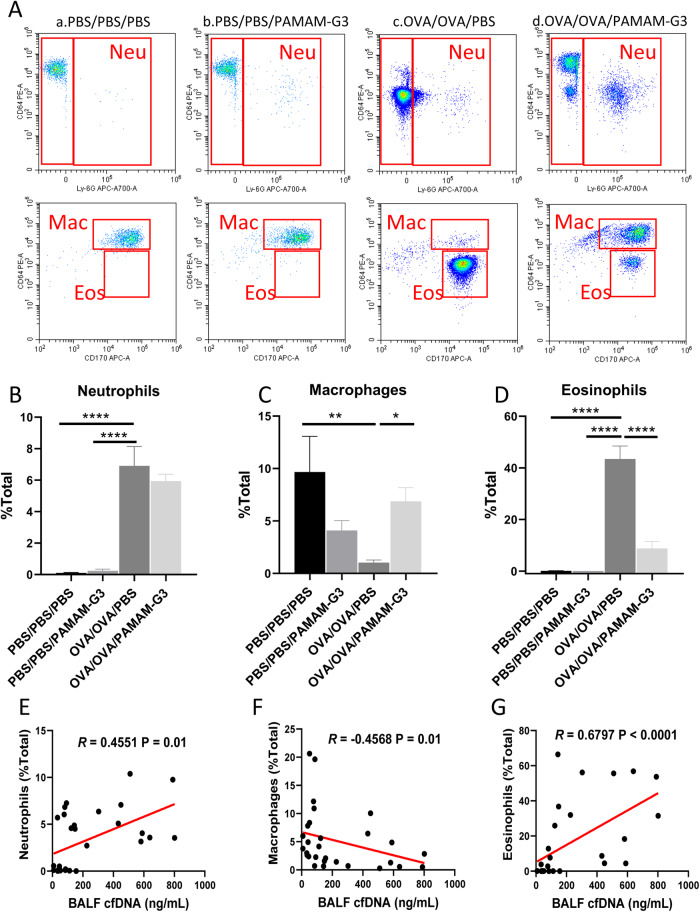
PAMAM-G3 reduced eosinophilic infiltration in AAI. Representative images (**A**) and the statistical analysis of the percentage of neutrophils (**B**)/macrophages (**C**) /eosinophils (**D**) in the total cell population. The correlation of neutrophils (Neu, **E**), macrophages (Mac, **F**), eosinophils (Eos, **G**) with cfDNA in BALF. Data was expressed as means ± SEM. *P < 0.05, **P < 0.01, ****P < 0.0001.

PAMAM-G3 showed a positive effect on scavenging cfDNA caused by allergen in vitro. To explore whether PAMAM-G3 has the same impact in vivo, we established an OVA-sensitized mouse model of AAI (Fig. 4A). Induction of AAI resulted in inflammatory cell infiltration in the peribronchial and perivascular tissues (Fig. 4B, D, *P* < 0.0001), and increased PAS-positive cells in bronchi (Fig. 4C, E, *P* < 0.0001). The levels of cfDNA (Fig. 5A, *P* < 0.0001), IL-4 (Fig. 5B, *P* = 0.02) and IL-13 (Fig. 5C, *P* = 0.01) in the BALF of OVA-sensitized mice were significantly higher than those in the control group. The levels of cfDNA were positively correlated with the concentrations of IL-4 (Fig. 5E, *R* = 0.5329, *P* < 0.0001) and IL-13 (Fig. 5F, *R* = 0.5634, *P* < 0.0001), respectively. The administration of intranasal PAMAM-G3 significantly reduced the inflammatory cell infiltration (Fig. 4B, D, *P* < 0.0001) and the PAS-positive cells in the lung (Fig. 4C, E, *P* = 0.003). Intranasal PAMAM-G3 suppressed the secretion of cfDNA (Fig. 5A, *P* = 0.0001), IL-4 (Fig. 5B, *P* = 0.02), and the ratio of IL-4 and IFN-γ (Fig. 5D, *P* = 0.0001) in the BALF compared to the OVA-sensitized mice treated with PBS. Meanwhile, intranasal PAMAM-G3 restored the proportion of macrophages (Fig. 6C, *P* = 0.03), and reduced the eosinophilic infiltration in the BALF (Fig. 6D, *P* < 0.0001). cfDNA levels in the BALF were correlated with the infiltration of inflammatory cells, including neutrophils, macrophages, and eosinophils, respectively (Fig. 6E–G).

## Discussion

Previous studies have suggested that cfDNA is involved in the mechanism of infection and autoimmune inflammation [36–40]. This study reveals for the first time that cfDNA plays a role in the pathogenesis of AR. Known as the united airway disease with AR [41], allergic asthma may be mediated by cfDNA, NETs, and EETs [21–23]. Our study contributes further evidence to support the shared underlying pathogenesis of the upper and lower allergic airway inflammation.

In this study, the correlation between cfDNA levels in EBC and Th2 cytokines indicated that the cfDNA levels in EBC were associated with the severity of allergic airway inflammation. EBC is a valuable non-invasive method for sampling airway biomarkers, particularly in children and the elderly [42, 43]. However, the limited detection of certain conventional cytokines in EBC restricts its applicability [42]. The measurement of cfDNA levels in EBC and nasal secretions has the potential to serve as a non-invasive objective biomarker for physicians to make dynamic adjustments, particularly in children with AR who frequently struggle to articulate or quantify symptoms and exhibit aversion to blood draws. However, it is imperative to validate the findings of this study in a pediatric population before implementing them in clinical practice.

To date, AIT is the only etiologic therapy for AAI. Nevertheless, a considerable proportion of AR patients, ranging from 30-40%, fail to derive benefits from AIT [44]. This lack of efficacy is often attributed to suboptimal adherence, which appears to be the prevailing factor underlying the inadequate response to treatment [45]. Our findings demonstrate a correlation between the duration of AIT and a reduction in cfDNA levels, thereby indicating the potential of cfDNA as a viable biomarker for assessing the response to AIT.

cfDNA is proven to be immunostimulatory when bound to carrier proteins or in oxidative states [46]. Our findings, which indicated that allergen exposure induced the release of cfDNA both in vitro and in vivo, suggest a potential role of cfDNA in the mechanism of AR. This is supported by previous research demonstrating that HDM challenge leads to DNA double-strand breaks and oxidative damage in vitro [47]. With the murine model of AAI, we revealed the increase of cfDNA in AAI and identified the association between cfDNA and the type 2 inflammation, consistent with the clinical data and in vitro findings. Several studies suggested that the application of DNase I could reduce the cfDNA in allergic asthma [20, 23]. However, it is important to note that DNase I has been associated with certain adverse effects, including voice changes and rash, particularly when administered via nebulized inhalation [48]. Besides, DNase I did not improve disease activity markers in SLE [46]. In this study, we utilized PAMAM-G3 to capture cfDNA according to the polyanion properties of DNA [49]. Our findings demonstrated that PAMAM-G3 effectively inhibited the secretion of cfDNA and diminishes allergic airway inflammation. Regarding the side effects, PAMAM-G3 is a relatively low generation of PAMAMs with relatively low cytotoxicity and also has a high efficacy in clearing cfDNA [50]. It’s reported that the in vivo application of PAMAMs can be eliminated within 2 h by kidney [50]. No significant systemic side effects have been observed in the intranasal application of PAMAM-G3 in a murine model [51]. The safety of its application in humans necessitates further meticulous evaluation. Engineered or targeted nanomaterial CPs have the potential to serve as an innovative and adaptable therapeutic approach for allergic diseases in general and AR specifically [28].

This study presents several potential implications. We demonstrated the potential role of cfDNA in the pathogenesis of AAI, although further research is needed to understand the underlying mechanism. While previous studies have linked NETs [20–22, 27] and EETs [22, 23] to allergic asthma, cfDNA contained numerous unidentified components. The utilization of DNA methylation signatures in cfDNA has been extensively employed for early diagnosis, prognosis prediction, and dynamic monitoring of cancers [52], as well as prenatal testing [53] and organ transplants [54]. Therefore, cfDNA has the potential to serve as an objective biomarker for AAI and warrants further investigation. Secondly, our study highlights the necessity for extensive cohort studies and prospective investigations to confirm the potential of cell-free DNA (cfDNA) as a biomarker for AIT. Thirdly, considering lipopolysaccharide (LPS) contamination in most HDM, LPS effect on cfDNA secretion was also evaluated (eFigure 3). Last but not least, our data reveals that HDM effect on cfDNA release is not limited to atopic nasal epithelial cells in the aspect of the abundance of cfDNA. However, further research is needed to determine if there is a specific composition of cfDNA associated with allergic airway inflammation.

In summary, this study shows that cfDNA is associated with allergic airway inflammation, and intranasal PAMAM-G3 could effectively scavenge cfDNA attenuating the AAI. These results provide a new perspective on the pathogenesis of AAI, and intranasal application of nanomedicine may offer a potential new strategy for AAI.

## Subject and methods

### Subjects

This study was approved by the Ethics Committee of the First Affiliated Hospital of Sun Yat-sen University (Approval No. 2022058) and all participants signed the informed consent. 54 healthy participants and 116 AR participants were recruited in this study. Characteristics of participants are listed in eTable 1 and eTable 2. AR was diagnosed based on their nasal and/or ocular hypersensitivity symptoms and specific immunoglobulin E (sIgE) tests according to the Allergic Rhinitis and its Impact on Asthma (ARIA) guidelines [55]. In this study, all AR participants were allergic to house dust mite (HDM). Total IgE (tIgE) and allergen specific IgE were evaluated according to the manufacturer’s instructions (HOB Biotech Group, MB00069). High tIgE group and normal tIgE group were divided by the age-dependent upper limit of tIgE levels [35]. HDM-sIgE grade was grouped by the serum concentration of HDM sIgE as follows: Grade 0 < 0.35 IU/mL, 0.35 ≤ Grade 1 < 0.7 IU/mL, 0.7 ≤ Grade 2 < 3.5 IU/mL, 3.5 ≤ Grade 3 < 17.5 IU/mL, 17.5 ≤ Grade 4 < 50 IU/mL, Grade 5 ≥ 50 IU/mL. AIT participants received AIT under the guidance of doctors. All healthy control (HC) participants had no airway symptom and had negative sIgE results. Exclusion criteria for all participants were as follows: (1) with chronic sinusitis, nasal polyps, asthma, atopic dermatitis, immunodeficiency or other diseases related to immune abnormality; (2) taking glucocorticoids and/or antihistamines within 2 weeks; (3) pregnancy; (4) uncooperative participants. Human exhaled breath condensate (EBC) samples were obtained by exhaled air condensate collector (Dingblue Technology BioscreenII, Beijing, China) and centrifuged at 350 × *g* for 5 min at 4 °C. Plasma samples were extracted from the peripheral blood with EDTA anticoagulation tube (BD, 367525) and centrifuged at 600 × *g* for 10 min at 4 °C. Nasal secretion samples were collected as previously described [56].

### Cell culture

BEAS-2B cells were acquired from American Type Culture Collection and cultured in DMEM nutrient mixture F12 medium supplemented with 10% fetal bovine serum (FBS) and 1% penicillin/streptomycin/L-glutamine (Gibco). Primary human nasal epithelial cells were obtained from 4 AR and 3 HC participants with sterile cytology brush (Puritan, 25-2199) and cultured in Complete PneumaCult-Ex Medium (Stemcell, 05008) with 0.1% Hydrocortisone Stock Solution and 1% penicillin/streptomycin/L-glutamine (Gibco). 2 × 10^4^ cells were cultured in 96 wells plates for 1 day and exposed to 50 μg/mL HDM (Greer, XPB91D3A2.5) with/without 15 μg/mL PAMAM dendrimers-Generation 3 (PAMAM-G3, Sigma-Aldrich, 412422) for 72 h. CpG ODN 684 (InvivoGen, tlrl-bw006) was used as the positive control for the secretion of cfDNA [18]. The supernatant was collected, centrifuged at 350 × *g* for 5 min at 4 °C, and stored at −80 °C. All cells were all tested and free from mycoplasma.

### Cell vitality test

Toxicity of PAMAM-G3 was assessed with Cell Counting Kit-8 (CCK-8, Yeasen, 40210ES10) according to the instructions. BEAS-2B cells were cultured in 96 wells plates for 1–2 days for cell multiplication. BEAS-2B cells were exposed to 50 μg/mL HDM (Greer, XPB91D3A2.5) with/without 15 μg/mL PAMAM-G3 for 72 h. Then, the medium was replaced with 100 μL CCK-8 solution for 2 h to stabilize the fluorescence, and measured optical density with microplate reader at 560 nm.

### Measurement of trans-epithelial electrical resistance (TEER)

3 × 10^5^ BEAS-2B cells were seeded onto Transwell inserts (area 0.33 cm^2^, pore size 0.4 µm; Costar, USA) and cultured in DMEM nutrient mixture F12 medium with 10% FBS and 1% penicillin/streptomycin/l-glutamine (Gibco) for 1 day for cell multiplication. Cells were exposed to 50 μg/mL HDM (Greer, XPB91D3A2.5) with/without 15 μg/mL PAMAM-G3 for 72 h. TEER was measured by EVOM™3 (WPI, USA) according to instructions.

### Extraction and measurement of cfDNA

To extract the cfDNA, the cellular media was centrifuged at 350 × *g* for 5 min at 4 °C to remove the cells. Then, the supernatant was used to extract cfDNA by using QIAamp MinElute ccfDNA Mini Kit (QIAGN, 55204). The concentrations of cfDNA were determined with QuantiT^TM^ PicoGreen^TM^ dsDNA Assay Kit (ThermoFisher, P7589) and Qubit dsDNA HS Assay Kit (ThermoFisher, Q32851).

### Animal experiments

BALB/c mice (female, 4–6 weeks of age) were purchased from Gempharmatech (Guangdong, China), and fed in a specific-pathogen-free (SPF) environment. This study was approved by the Institutional Animal Care and Use Committee, Sun Yat-sen University (No.2021749). Ovalbumin (OVA) was used as the allergen in this murine model and phosphate buffered saline (PBS) was used as the media control. As shown in Fig. 4A, mice were divided into 4 groups and named by sensitization/challenge/treatment as follows: PBS/PBS/PBS, PBS/PBS/PAMAM-G3, OVA/OVA/PBS, OVA/OVA/PAMAM-G3. To build the mouse model of AAI, BALB/c mice were intraperitoneal sensitized with 40 μg OVA (Sigma-Aldrich, A5503) in 100 μL PBS emulsified with 100 μL of alum adjuvant (ThermoFisher, 77161-50 ML) every 7 days, 3 times in total. From day 21 to 28, BALB/c mice were daily challenged with intranasally infused with 20 μL OVA (25 mg/mL) and immediately aerosolized with 5% OVA (0.5 g OVA/10 mL PBS) through an air-compressing nebulizer (Yuyue 403 A, Jiangsu, China) for 30 min. PBS and PAMAM-G3 (2 mg/kg) were intranasally given to corresponding groups after aerosolization. BALB/c mice were euthanized and sampled at 4 h post challenged. After the execution, BALF was collected with 1 mL 4 °C PBS, centrifuged at 2000 rpm for 10 min at 4 °C. The supernatant was stored at −80 °C. BALF cells were collected for flow cytometry. Lung tissues were soaked in 4% paraformaldehyde for 48 h, paraffin-embedded and stained with hematoxylin and eosin (H&E) and periodic acid-Schiff (PAS). Inflammation were measured as previously described [57]. Double blind was used during inflammation evaluation.

### Enzyme-linked immunosorbent assay (ELISA)

Human EBC and plasma samples were collected and analyzed by using anti-human Interleukin (IL)-4 (ThermoFisher, 88-7046-77) and anti-human IL-5 ELISA kits (ThermoFisher, 88-7056-88), according to the manufacturer’s instructions. Mouse BALF samples were centrifuged at 2000 rpm for 10 min at 4 °C and analyzed with anti-mouse IL-4 (ThermoFisher, 88-7044-88), anti-mouse IL-5 (ThermoFisher, 88-7314-88), anti-mouse IL-13 (ThermoFisher, 88-7137-88) and anti-mouse IFN-gamma (ThermoFisher, 88-7314-88) ELISA kits according to the instructions.

### Flow cytometry

BALF cells were collected as mentioned above. After being washed and processed with red blood cell lysis buffer, BALF cells were incubated with APC anti-mouse CD170 (Biolegend, 155508), APC/Cyanine7 anti-mouse CD11b (Biolegend, 101226), PE/Cyanine7 anti-mouse Ly-6C (Biolegend, 128018), FITC anti-mouse CD45 (Biolegend, 103108), Alexa Fluor® 700 anti-mouse Ly-6G (Biolegend, 127622) and PE anti-mouse CD64 (Biolegend, 139304) for 30 min. Then BALF cells were analyzed on a CytoFLEX flow cytometer (Beckman Coulter). The gating strategies are shown in eFigure 1.

### Statistical analysis

Results were expressed as means ± SEM (mainly shown in eTable 3). Data were analyzed with GraphPad Prism 8 software (La Jolla, California, USA). T tests were used to analyze 2-group comparison if the data fit Gaussian distribution, if not, Mann-Whitney tests were conducted. Multiple comparisons were conducted with Ordinary one-way ANOVA if the data fit Gaussian distribution, otherwise, Friedman tests were conducted. When *P* values were less than 0.05, we consider it statistically significant.

### Supplementary information

Supplementary Material

## Data Availability

The corresponding author will provide the original data used to support the findings of this study upon reasonable request.
