# Supplementary material for: Intranasal PAMAM-G3 scavenges cell-free DNA attenuating the allergic airway inflammation
Source: Cell Death Discov. 2024 May 2;10:213. doi: 10.1038/s41420-024-01980-x (PMC11065999; doi:10.1038/s41420-024-01980-x)
Supplement: Supplementary file 1 — Supplementary Material [file 41420_2024_1980_MOESM1_ESM.docx]

Supplementary Material

**eTable 1** **Characteristics of study participants of plasma and exhaled breath condensate**

| **Characteristics** | **Healthy control** | **Allergic rhinitis** | **Allergen**  **immunotherapy** |
| --- | --- | --- | --- |
| **Sample Size** | 54 | 116 | 39 |
| **Age, year** | 25.41±0.6197 | 30.69±0.8536 | 22.41±2.038 |
| **Sex**  **Female，n(%)**  **Male，n(%)** | 30 (55)  24 (45) | 51 (44)  65 (56) | 17(44)  22(56) |
| **Serum total IgE, IU/mL**  **Mite specific IgE,** **IU/mL** | 114.6±15.80  0.08444±0.008108 | 271.0±16.22  45.98±5.892 | - |
| **Treatment time, day** | - | - | 401.2±43.07 |
| **VAS**  **Stuffiness**  **Itchy nose**  **Sneezing**  **Running nose**  **Itchy eyes**  **Eye foreign body sensation**  **Red eyes**  **Watery eyes**  **Wheezing**  **Cough**  **Dyspnea**  **Chest tightness**  **Overall** | - | 5.548±0.2418  4.739±0.2327  6.235±0.2514  6.113±0.2599  3.557±0.2782  2.183±0.2403  2.052±0.2445  2.087±0.2428  0.9826±0.1747  1.113±0.1905  0.7478±0.1481  0.6696±0.1379  4.374±0.2351 | 3.013±0.3458  1.577±0.2690  3.064±0.3064  2.372±0.3105  1.308±0.3185  -  -  0.2179±0.09856  -  -  -  -  - |

**eTable 2 Characteristics of study participants of nasal secretion**

| **Characteristics** | **Healthy control** | **Allergic rhinitis** |
| --- | --- | --- |
| **Sample Size** | 26 | 23 |
| **Age, year** | 23.81±0.4507 | 32.39±1.590 |
| **Sex**  **Female，n(%)**  **Male，n(%)** | 14(52)  13(48) | 13(57)  10(43) |
| **Serum total IgE, IU/mL**  **Mite specific IgE,** **IU/mL** | 95.18±12.32  0.1385±0.01762 | 232.5±32.56  30.80±10.36 |
| **VAS**  **Stuffiness**  **Itchy nose**  **Sneezing**  **Running nose**  **Itchy eyes**  **Eye foreign body sensation**  **Red eyes**  **Watery eyes**  **Wheezing**  **Cough**  **Dyspnea**  **Chest tightness**  **Overall** | - | 5.348±0.5053  5.087±0.6060  6.391±0.5543  6.522±0.5024  3.348±0.6147  1.478±0.4164  1.870±0.5119  1.696±0.4723  0.6957±0.2699  0.5652±0.2069  0.4783±0.2073  0.6957±0.2699  4.913±0.4118 |

**eTable 3 Statistical analysis results**

| **Figure** | **Groups** | **Sample Size** | **Mean±SEM** | ***P* value** |
| --- | --- | --- | --- | --- |
| Figure 1A | Plasma  HC vs AR | 54 vs 111 | 347.7±20.99 vs 432.5± 17.66 | 0.003 |
| Figure 1B | Nasal secretion  HC vs AR | 24 vs 20 | 305.8±81.99 vs 585.9±123.7 | 0.02 |
| Figure 1C | EBC  HC vs Grade 5 | 54 vs 35 | 67.70±16.25 vs 168.1± 34.62 | 0.01 |
| Figure 1D | Nasal secretion  normal tIgE vs high tIgE | 20 vs 24 | 316.3±97.39 vs 530.4±106.6 | 0.048 |
| Figure 1E | EBC  normal tIgE vs high tIgE | 48 vs 65 | 64.53±14.79 vs 137.2±24.68 | 0.02 |
| Figure 2 | Media vs HDM 50μg/mL | 9 vs 9 | 71.92±6.446 vs 125.5±12.80 | 0.0004 |
| Figure 2 | Media vs CpG DNA 2μg/mL | 9 vs 9 | 71.92±6.446 vs 234.4±4.001 | <0.0001 |
| Figure 3B | Media vs HDM 50μg/mL | 9 vs 9 | 388.2±5.834 vs 550.1±15.97 | <0.0001 |
| Figure 3B | HDM 50μg/mL vs  HDM 50μg/mL+PAMAM-G3 15μg/mL | 9 vs 9 | 550.1±15.97 vs 488.9±7.934 | <0.0001 |
| Figure 3C | Media vs HDM 50μg/mL | 6 vs 6 | 16.17±0.8647 vs 11.94±0.6050 | 0.0004 |
| Figure 3C | HDM 50μg/mL vs  HDM 50μg/mL+PAMAM-G3 15μg/mL | 6 vs 6 | 11.94±0.6050 vs 14.63±0.3581 | 0.02 |
| Figure 3D | Media vs HDM 50μg/mL | 8 vs 8 | 100.0±0.000 vs 17.38±2.117 | <0.0001 |
| Figure 3D | HDM 50μg/mL vs  HDM 50μg/mL+PAMAM-G3 15μg/mL | 8 vs 8 | 17.38±2.117 vs 41.92±2.483 | 0.0002 |
| Figure 3F | HC: Media vs HDM 50μg/mL | 10 vs 8 | 80.145±17.987 vs 644.056±33.160 | <0.0001 |
| Figure 3F | HC: HDM 50μg/mL vs  HDM 50μg/mL+PAMAM-G3 15μg/mL | 8 vs 8 | 644.056±33.160 vs 89.264±20.996 | <0.0001 |
| Figure 3F | AR: Media vs HDM 50μg/mL | 11 vs 12 | 136.232±21.617 vs 759.572±47.060 | <0.0001 |
| Figure 3F | AR: HDM 50μg/mL vs  HDM 50μg/mL+PAMAM-G3 15μg/mL | 12 vs 12 | 759.572±47.060 vs 107.964±15.079 | <0.0001 |
| Figure 3F | HC HDM 50μg/mL vs AR HDM 50μg/mL | 8 vs 12 | 644.056±33.160 vs 759.572±47.060 | 0.01 |
| Figure 3G | HC: Media vs HDM 50μg/mL | 10 vs 8 | 100.00±1.093 vs 67.432±8.276 | 0.001 |
| Figure 3G | HC: HDM 50μg/mL vs  HDM 50μg/mL+PAMAM-G3 15μg/mL | 8 vs 8 | 67.432±8.276 vs 98.457±3.283 | 0.003 |
| Figure 3G | AR: Media vs HDM 50μg/mL | 11 vs 12 | 100.00±1.034 vs 42.394±7.207 | <0.0001 |
| Figure 3G | AR: HDM 50μg/mL vs  HDM 50μg/mL+PAMAM-G3 15μg/mL | 12 vs 12 | 42.394±7.207 vs  68.243±8.554 | 0.002 |
| Figure 3G | HC HDM 50μg/mL vs AR HDM 50μg/mL | 8 vs 12 | 67.432±8.276 vs 42.394±7.207 | 0.01 |
| Figure 3G | HC HDM+PAMAM-G3 15μg/mL vs AR HDM+PAMAM-G3 15μg/mL | 8 vs 12 | 98.457±3.283 vs 68.243±8.554 | 0.002 |
| Figure 4B | PBS/PBS/PBS vs OVA/OVA/PBS | 12 vs 15 | 0.08333±0.05981 vs 2.622±0.08524 | <0.0001 |
| Figure 4B | PBS/PBS/PAMAM-G3 vs OVA/OVA/PBS | 12 vs 15 | 0.3611±0.07630 vs 2.622±0.08524 | <0.0001 |
| Figure 4B | OVA/OVA/PBS vs OVA/OVA/PAMAM-G3 | 15 vs 15 | 2.622±0.08524 vs 1.467±0.1117 | <0.0001 |
| Figure 4C | PBS/PBS/PBS vs OVA/OVA/PBS | 12 vs 15 | 0.000±0.000 vs 42.89±9.529 | <0.0001 |
| Figure 4C | PBS/PBS/PAMAM-G3 vs OVA/OVA/PBS | 12 vs 15 | 0.000±0.000 vs 42.89±09.529 | <0.0001 |
| Figure 4C | OVA/OVA/PBS vs OVA/OVA/PAMAM-G3 | 15 vs 15 | 42.89±9.529 vs 16.51±3.478 | 0.003 |
| Figure 5A | PBS/PBS/PBS vs OVA/OVA/PBS | 12 vs 15 | 81.21±17.94 vs 407.8±59.23 | <0.0001 |
| Figure 5A | PBS/PBS/PAMAM-G3 vs OVA/OVA/PBS | 12 vs 15 | 37.96±11.85 vs 407.8±59.23 | <0.0001 |
| Figure 5A | OVA/OVA/PBS vs OVA/OVA/PAMAM-G3 | 15 vs 15 | 407.8±59.23 vs 144.4±47.77 | 0.0001 |
| Figure 5B | PBS/PBS/PBS vs OVA/OVA/PBS | 12 vs 15 | 88.75±21.71 vs 283.5±80.49 | 0.02 |
| Figure 5B | PBS/PBS/PAMAM-G3 vs OVA/OVA/PBS | 12 vs 15 | 55.32±13.31 vs 283.5±80.49 | 0.005 |
| Figure 5B | OVA/OVA/PBS vs OVA/OVA/PAMAM-G3 | 15 vs 15 | 283.5±80.49 vs 105.5±23.06 | 0.02 |
| Figure 5C | PBS/PBS/PBS vs OVA/OVA/PBS | 12 vs 15 | 3.906±0.000 vs 40.60±14.85 | 0.01 |
| Figure 5C | PBS/PBS/PAMAM-G3 vs OVA/OVA/PBS | 12 vs 15 | 3.906±0.000 vs 40.60±14.85 | 0.01 |
| Figure 5C | OVA/OVA/PBS vs OVA/OVA/PAMAM-G3 | 15 vs 15 | 40.60±14.85 vs 15.28±4.643 | 0.09 |
| Figure 5D | PBS/PBS/PBS vs OVA/OVA/PBS | 12 vs 15 | 4.434±1.022 vs 27.57±5.044 | <0.0001 |
| Figure 5D | PBS/PBS/PAMAM-G3 vs OVA/OVA/PBS | 12 vs 15 | 5.163±1.297 vs 27.57±5.044 | <0.0001 |
| Figure 5D | OVA/OVA/PBS vs OVA/OVA/PAMAM-G3 | 15 vs 15 | 27.57±5.044 vs 9.380±1.351 | 0.0001 |
| Figure 6B | PBS/PBS/PBS vs OVA/OVA/PBS | 6 vs 9 | 0.1000±0.03077 vs 6.903±1.237 | <0.0001 |
| Figure 6B | PBS/PBS/PAMAM-G3 vs OVA/OVA/PBS | 6 vs 9 | 0.2383±0.1069 vs 6.903±1.237 | <0.0001 |
| Figure 6B | OVA/OVA/PBS vs OVA/OVA/PAMAM-G3 | 9 vs 8 | 6.903±1.237 vs 5.940±0.4277 | 0.7 |
| Figure 6C | PBS/PBS/PBS vs OVA/OVA/PBS | 6 vs 9 | 9.663±3.410 vs 1.029±0.2486 | 0.003 |
| Figure 6C | PBS/PBS/PAMAM-G3 vs OVA/OVA/PBS | 6 vs 9 | 4.092±0.9380 vs 1.029±0.2486 | 0.4 |
| Figure 6C | OVA/OVA/PBS vs OVA/OVA/PAMAM-G3 | 9 vs 8 | 1.029±0.2486 vs 6.868±1.309 | 0.03 |
| Figure 6D | PBS/PBS/PBS vs OVA/OVA/PBS | 6 vs 9 | 0.1033±0.03490 vs 43.48±5.003 | <0.0001 |
| Figure 6D | PBS/PBS/PAMAM-G3 vs OVA/OVA/PBS | 6 vs 9 | 0.04667±0.01054 vs 43.48±5.003 | <0.0001 |
| Figure 6D | OVA/OVA/PBS vs OVA/OVA/PAMAM-G3 | 9 vs 8 | 43.48±5.003 vs 8.809±2.692 | <0.0001 |
| eFigure 3 | Media vs HDM 50μg/mL | 7 vs 7 | 108.6±2.068 vs 433.6±39.84 | <0.0001 |
| eFigure 3 | Media vs Derp 1 3.825μg/mL | 7 vs 7 | 108.6±2.068 vs 399.4±44.75 | <0.0001 |
| eFigure 3 | Media vs LPS | 7 vs 7 | 108.6±2.068 vs 247.0±25.07 | 0.005 |
| eFigure 3 | HDM 50μg/mL vs Derp1 3.825 μg/mL | 7 vs 7 | 433.6±39.84 vs 399.4±44.75 | 0.8 |
| eFigure 3 | HDM 50μg/mL vs LPS 0.5ng/mL | 7 vs 7 | 433.6±39.84 vs 247.0±25.07 | 0.0001 |
| eFigure 3 | HDM 50μg/mL vs HDM 50μg/mL+PAMAM-G3 15μg/mL | 7 vs 7 | 433.6±39.84 vs 185.2±16.08 | <0.0001 |

**Supplementary Figures**

**
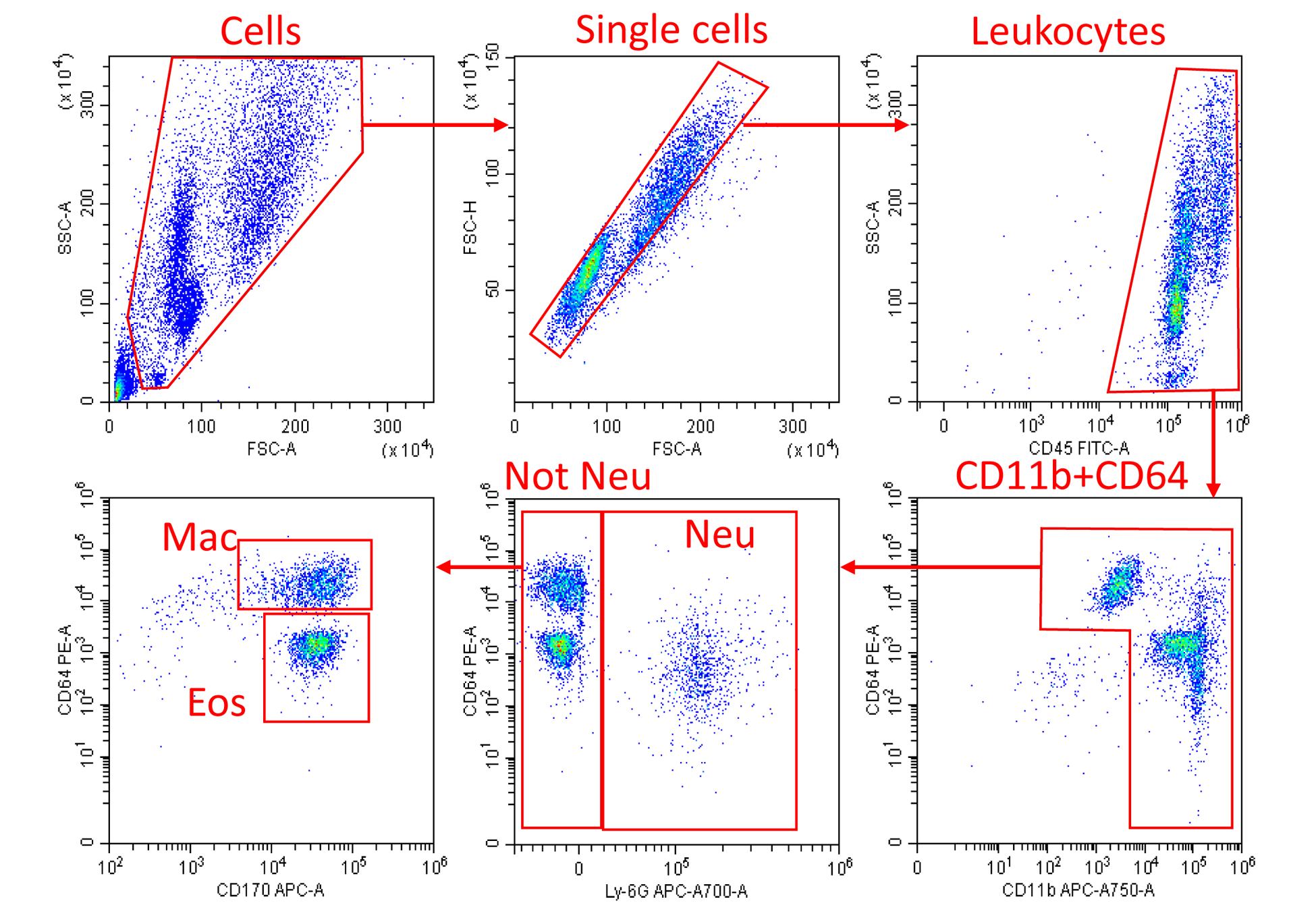
**

**eFigure 1.** **The gating strategies of flow cytometry analyses for the bronchoalveolar lavage fluid in the murine model of allergic airway inflammation.** Neu, neutrophils; Mac, macrophages; Eos, eosinophils.

**eFigure 2.** **The correlation analysis between the concentrations of HDM-sIgE in plasma and the cfDNA levels in the exhaled breath condensate (EBC) from the allergic rhinitis participants.** HDM, house dust mite; sIgE, specific immunoglobulin E; EBC, exhaled breath condensate; cfDNA, cell-free DNA.

**eFigure 3 Effect of different components of House dust mite (HDM, Greer, XPB91D3A2.5, Lot Number: 406945) on cfDNA secretion.** According to the certificate of analysis of HDM, 50 μg/mL HDM contains 3.825 μg/mL Derp1 (Indoor ,LTN-DP1-1) and 0.5 ng/mL Lipopolysaccharide (LPS, Sigma-Aldrich, L4516) at most. PAMAM-G3, Polyamidoamine dendrimers generation 3. Data was expressed as means ± SEM. **P < 0.01, ***P < 0.001, ****P < 0.0001.
